# Larregue’s Critique of Cofnas et al. (2017): A Rejoinder

**DOI:** 10.1007/s12108-018-9372-6

**Published:** 2018-03-12

**Authors:** Nathan Cofnas, Noah Carl, Michael A. Woodley of Menie

**Affiliations:** 1 Balliol College, Oxford, OX1 3BJ UK; 2 Nuffield College, Oxford, UK; 3 Center Leo Apostel for Interdisciplinary Studies, Vrije Universiteit Brussel, Brussels, Belgium

**Keywords:** Politics and science, Trust in science, Political polarization, Political bias in science

## Abstract

Data from the General Social Survey indicate that conservatives’ self-reported trust in scientists has steadily decreased since 1974. In Cofnas et al. (*The American Sociologist*, 2017), we suggested that this trend may have been partly driven by the increasing tendency of scientific institutions, and the representatives of such institutions, to distort social science for the sake of liberal activism. Larregue (*The American Sociologist*, 2017) makes three opposing arguments: (1) It is “very hard” to establish the charge of bias, especially since we did “not state what [we] mean by ‘bias.’” (2) We did not establish a causal relationship between scientists’ (alleged) liberal activism and conservatives’ distrust of science, and we ignored activism by conservative scientists. (3) We were wrong to advocate “affirmative action” for conservatives in academia. We address these arguments in turn: (1) Larregue does not engage with our main arguments that liberal bias exists in social science. (2) In recent years, prominent scientific organizations have, with great publicity, intervened in policy debates, always supporting the liberal side without exception. It is not unreasonable to assume that this would diminish conservatives’ trust in these organizations. Contra Larregue, in Cofnas et al. (*The American Sociologist*, 2017) we explicitly acknowledged that conservative scientists can also be biased. (3) We never advocated “affirmative action” for conservatives, and in fact we object to such a proposal.

## Introduction

Based on data from the General Social Survey (GSS), Gauchat (2012) reports that American conservatives’ trust in science has fallen significantly since 1974. GSS interviewers preface their question about trust in science with the following:

I am going to name some institutions in this country. As far as the people running these institutions are concerned, would you say you have a great deal of confidence, only some confidence, or hardly any confidence at all in them?

The interviewer then asks respondents about “the Scientific Community.” In 1974, 49% of self-identified conservatives responded with “a great deal” compared with 48% of liberals and 45% of moderates. Moderates started reporting less confidence in the late 70s and early 80s. Conservatives’ confidence dropped steadily, while liberals showed no discernible change. By 2010, 38% of conservatives, 40% of moderates, and 50% of liberals answered “a great deal.”

In Cofnas et al. (2017), we suggested that declining conservative trust in scientists—or the leaders of scientific institutions—might be due to increasing liberal activism by scientific institutions. We provided several examples where prominent, mainstream scientific organizations or prominent members of the scientific community have misrepresented findings in science (e.g., in testimony to Congress or as *amici curiae*) to promote liberal causes. We cited evidence that conservatives have lost trust not in all scientists, but only in so-called “impact scientists,” i.e., those whose work has policy implications (e.g., psychologists who study discrimination). Conservatives have *greater* trust than liberals in “production scientists” whose work does not have policy implications (e.g., agricultural scientists) (McCright et al. 2013). In Cofnas et al. (2017), we focused on activism in social science, which encompasses the paradigm “impact sciences” (sociology and psychology) that have played a role in numerous political debates in the last few decades.

Larregue (2017) makes three opposing arguments to our paper: (1) It is “very hard” to establish the charge of bias, especially since we did “not state what [we] mean by ‘bias.’” (2) We did not establish a causal relationship between scientists’ (alleged) liberal activism and conservatives’ distrust of science, and we ignored activism by conservative scientists. (3) We were wrong to advocate “affirmative action” for conservatives in academia. We address these arguments in turn: (1) Larregue does not engage with our main arguments that liberal bias exists in social science. (2) In recent years, prominent scientific organizations have, with great publicity, intervened in policy debates, always supporting the liberal side without exception. It is not unreasonable to assume that this would diminish conservatives’ trust in these organizations. Contra Larregue, in Cofnas et al. (2017) we explicitly acknowledged that conservative scientists can also be biased. (3) We never advocated “affirmative action” for conservatives, and in fact we object to such a proposal.

## Is There a Liberal Bias in Social Science?

Citing Gross (2013), Larregue (2017) suggests that scholars in different fields have different views about the extent to which “politics should influence teaching and research.” Those in some disciplines, such as biology, tend to take an “objectivist stance,” denying that politics should have any influence on research. Scholars in other disciplines, such as the humanities, are “much more skeptical about professors’ capacity to keep politics at bay” (i.e., they accept that researchers’ politics can legitimately influence their work). Larregue writes:

As Neil Gross found in the interviews he conducted, “sociology is best described as an epistemological hybrid, combining elements of objectivism and skepticism” (Gross 2013, p. 190). Interestingly, sociologists were further fractally divided between two scientific poles: those closer to the humanities or activist camp, who shared the belief that objectivism is an illusion, and those who endorsed a more professional and positivistic stance, for whom sociology should try to attain objectivity. This latter epistemological position was dominant among the sociologists interviewed by Gross: “For most American sociologists, in other words, objectivity remains an ideal toward which they claim to strive, even as they recognize the impossibility of ever fully achieving it” (Gross 2013, p. 200). This tends to contradict CCW’s [i.e., Cofnas et al.’s] claim that social scientists are consciously distorting their science to fit their agenda.

There are a couple things to say about this. First, according to the source that Larregue himself seems to take as authoritative, the “best descri[ption]” of sociology is that it is an “epistemological hybrid” that is only partly committed to “objectivism.” This suggests that, at the very least, there is a major element within sociology that does not even *try* to “keep politics at bay.” Second, Larregue cites Gross’s report that “most American sociologists” that he (Gross) interviewed endorsed the objectivist stance. According to Larregue, “This tends to contradict [our] claim that social scientists are consciously distorting their science to fit their agenda.” But what does it mean for this to “contradict” our claim? In light of the evidence we provided—i.e., examples of large numbers of social scientists acting collectively through their principal organizations to misrepresent science—how much probative value does Gross’s survey have? The fact that more than 50% of sociologists say in a survey that they are committed to objectivism does not, in our view, overturn strong evidence, based on their *behavior*, that they often distort science for political reasons.

Larregue argues:

To substantiate their claims, CCW use cherry-picked examples that, of course, confirm their hypothesis of a liberal bias in science. One major problem with CCW’s argument is that they do not clearly state what they mean by bias. Since we can envision more or less strict or lenient conceptions of what constitute a bias in science, their statement is quasi-irrefutable.

The claim that we “cherry-picked” examples implies that there where comparable examples of both liberal *and* conservative bias, and we cited only examples of the former. So if we were guilty of cherry-picking, this would be very easy to demonstrate. Larregue simply needed to show several examples from the past few decades of a major, mainstream social science organization (such as the American Sociological Association or the American Psychological Association), or a recognized leader of a social scientific field (such as Robert Putnam), distorting science to advance a *conservative* political cause. But as far as we know not a single example of conservative bias like that exists at all—Larregue certainly did not mention one. There may well be individual social scientists who are biased in favor of conservative-friendly interpretations of science, or right-wing think tanks that distort science. But these individuals and partisan think tanks have no influence over the major scientific organizations that represent the scientific establishment as a whole, and conservative mavericks are not regarded as leaders of their fields.

Larregue is in a way correct when he says that we “mix up different types of bias in the examples [we] use,” such as “fraud” and “advocacy.” But we believe he draws an incorrect conclusion from this fact when he claims that this “contributes to blur [our] demonstration.” Rather, our examples just show that mainstream social scientists engage in several different types of bias to advance liberal causes. The fact that some of our examples reveal multiple types of (liberal) bias operating simultaneously does not undermine our thesis that there is liberal bias in social science. Our thesis is not “quasi-irrefutable” because, to repeat, it would be refuted if Larregue could show examples of any type of bias employed by a mainstream social scientific organization to advance a conservative political cause.

Larregue says that to “substantiate” our claims, we “rely on” Inbar and Lammer’s (2012) “survey of social and personality psychologists that showed liberal scientists willing to discriminate against their conservative colleagues.” But, he says:

When they come to the implications of their results, Yoel Inbar and Joris Lammers do not mention any sort of liberal bias that would distort research to fit political agendas. Their only statement regarding the content of science is that many aspects of conservative thinking can serve as inspiration for interesting research questions that would otherwise be missed (Inbar and Lammers 2012, p. 502). Apart from the fact that this is an unsubstantiated supposition, this argument by no means implies that liberal science is biased or wrong. Here again the authors confuse between the context of pursuit of research with the context of justification.

Here Larregue is criticizing Inbar and Lammers’s argument—which we do not refer to at all—and portraying it as a criticism of *our* argument, which is different. As we will discuss more below, we do *not* necessarily believe that “conservative thinking can serve as inspiration for interesting research questions that would otherwise be missed.” But we do suggest that Inbar and Lammers’s findings reflect the existence of a liberal establishment in social science that seeks to suppress ideas that are not friendly to liberal politics. If a large percentage of personality and social psychologists admit that they would be inclined to reject conservative-friendly papers in peer review, to not invite a known political conservative to a symposium, and to reject conservative grant applications and job applications, this would contribute to an environment that is not receptive to liberal-undermining ideas, even if those ideas are sometimes correct.

## The Causal Link Between Scientists’ Liberal Activism and Conservatives’ Distrust, and the Issue of Conservative Activism

### Measuring Activism

Larregue raises the problem of how we can show a causal relationship between increasing activism among scientists and conservatives’ distrust. How can we measure an increase in activism? “What variables can account for scientists’ social activism over time? CCW give no answer to these questions. They give examples of what they interpret as political activism, sure, but this does not prove a statistical increase.”

It is true that activism among scientists is difficult to measure. There is no uncontroversial way to count up instances of activism among scientists, and measure a quantitative increase over the last four decades. However, just because we cannot investigate this question using ideal quantitative methods does not mean that the question cannot be approached in a reasonably systematic and scientific way. A great deal of historical analysis faces the same methodological problem. We can only point to numerous examples where scientists appear to have engaged in liberal activism in recent years. Since neither Larregue nor anyone else has been able to give comparable examples of conservative activism among scientists in the past few decades, we think it is reasonable to conclude that there is a trend toward more liberal activism.

It is difficult to prove that there is a *causal* link between increasing liberal activism and conservative distrust in science. But we think it is a reasonable explanation for decreasing conservative trust in social science that, in the course of practically all major political debates, representatives of the social science community make an appearance to promote research that supports the liberal side. Whether it’s gun control, death penalty cases, affirmative action, or anything else, the ASA or the APA or the American Political Science Association—or sometimes all these organizations together—will testify to congress or submit *amicus curiae* briefs arguing that “science” shows that the liberals are right. It seems the onus is on the critic to show why this *wouldn’t* decrease conservatives’ trust in social science.

### Did We Ignore Conservative Bias?

Larregue claims that “As conservative apostles of objectivity, CCW believe that liberal scientists are necessarily biased. Conversely, the mere possibility that conservative thinkers might be, too, biased, is not even mentioned.” This is false. In Cofnas et al. (2017), we say, based on Honeycutt and Freberg’s (2017) findings, that “The same proportion of conservatives and liberals [in academia] expressed willingness to discriminate against those with opposing political views.” We calculated that about 1.33% of personality and social psychologists are conservatives who would acknowledge that they would discriminate against liberals. We then noted that a journal submission that goes through an editor and three referees would have a 5% chance of being handled by a conservative who discriminates against liberals. So we clearly acknowledged that conservatives can be biased, and we portrayed this as a problem.

Larregue repeatedly accuses us of failing to recognize the difference between the “context of discovery” and the “context of justification,” because we supposedly assume that if scientists undertake scientific investigation with a liberal agenda then their results must be false. Larregue notes that it is possible to be motivated by bias but make legitimate discoveries. Ironically, however, it is Larregue who repeatedly confuses the contexts of discovery and justification in his criticisms of us and of conservatives. For example, he says:

CCW also fail to take into consideration conservative scientists’ activism....In intelligence and race research, conservative scientists, from Arthur Jensen to Richard Herrnstein and Charles Murray (authors of The Bell Curve), were partly financed by the Pioneer fund, a conservative organization that was active in the American eugenic movement (Kühl 2002).

First of all, Richard Herrnstein and Charles Murray never received funding from the Pioneer Fund. (They were criticized for *citing* people who had received such funding.) More important, the fact that science is funded by an organization with a political agenda does not mean that the funded science itself is ipso facto “activism.”

In Cofnas et al., we never claimed that liberal scientists engaged in activism simply because they received funding from liberal organizations. The question is whether prominent, mainstream scientists and scientific organizations actively distort science to advance a political agenda. *That* is what we provided evidence for.

Larregue also attempts to discredit us by claiming that *we*—Cofnas, Carl, and Woodley of Menie—are motivated to criticize liberal bias in social science by our conservative agenda to “advance the position of conservative scientists within academia.” Besides the fact that this is an illegitimate criticism because it conflates the contexts of discovery and justification, the evidence that Larregue brings forward to prove our commitment to a conservative agenda is problematic—even, we might say, biased. For example, he says:

The lead author, Nathan Cofnas, has published op-eds in conservative outlets, including *National Review* and *Quillette*. Recently, he accused Stephen Hawking of promoting a “Dadaist science”, after the world-renowned physicist said that Trump’s decision to withdraw from the Paris climate accord would “push the Earth over the brink.”

This is inaccurate. Cofnas (2017) criticized Hawking for his claim that Trump’s withdrawal from the Paris climate accord would “push the Earth over the brink *to become like Venus, with a temperature of two hundred and fifty degrees [Celsius], and raining sulphuric acid.*” As a Google search of that quote will reveal, Hawking was widely criticized by mainstream climate scientists for expressing a view that had no justification whatsoever. Larregue accuses Noah Carl of having a conservative agenda because he “has contributed to the conservative academic blog *Heterodox Academy*.” The fact that Larregue sees Heterodox Academy as “conservative” is rather illustrative, given that the organization is nonpartisan in both its mission statement and the composition of its members. As of June 26, 2017, Heterodox Academy’s members identified as Left/Progressive (18.2%), Right/Conservative (17.3%), Centrist/Moderate (24.9%), Libertarian/Classical Liberal (23.2%). (16.4% were unclassifiable, preferred not to say, or were “other.”). Regarding Woodley of Menie’s supposed bias, Larregue says: “Michael A. Woodley of Menie...has given an interview about human intelligence to Stefan Molyneux, a well-known Canadian conservative podcaster who supported Trump's campaign.” But Molyneux has conducted interviews on human intelligence with outspoken liberals including Eric Turkheimer and James Flynn. The fact that Molyneux interviewed someone does not indicate that he is a conservative, let alone that he has a conservative bias.^1^

### Should There Be “Affirmative Action” for Conservatives in Social Science?

As discussed, Larregue devotes one-out-of-three of the main sections of his paper to criticizing us for advocating affirmative action for conservatives. He says: “The third section of this article is devoted to CCW’s political proposal, namely introducing ‘affirmative action’ for conservatives in academia.” Note the quotes he puts around the phrase “affirmative action,” which makes it seem that he is quoting us. However, the words “affirmative action” do not appear anywhere in Cofnas et al. (2017). We suspect that Larregue misinterpreted the last paragraph of our paper:

In the past few years, a number of social scientists, led by Jonathan Haidt, have called upon social scientists to diversify the field and make a conscious effort to root out liberal bias (Duarte et al. 2015). We conclude with a prediction: If social scientists begin counteracting liberal activism, the trend of lowering conservative trust in scientists will reverse. (Cofnas et al. 2017)

In this paragraph, we refer to the fact that Haidt endorses active efforts to recruit conservatives to social science, but we do not endorse this proposal. In fact we do not think it is a good idea. Our personal hope is that scientists, whether they are liberal or conservative, will stop being biased and discriminating against others for political reasons. If this happens, we think more conservatives will become social scientists because it will be much easier for them to be hired and to pursue research that may not support liberal assumptions about how the world works.

## Footnotes

1 The results of this survey can be found on Heterodox Academy’s website (http://heterodoxacademy.org/krugman-is-wrong-we-are-not-conservative/).
